# Seroprevalence of Hepatitis E Virus Infection among Swine Farmers and the General Population in Rural Taiwan

**DOI:** 10.1371/journal.pone.0067180

**Published:** 2013-06-26

**Authors:** Jian-Te Lee, Pei-Lan Shao, Luan-Yin Chang, Ning-Shao Xia, Pei-Jer Chen, Chun-Yi Lu, Li-Min Huang

**Affiliations:** 1 Department of Pediatrics, National Taiwan University Hospital, Yun-Lin Branch, Yunlin, Taiwan; 2 Department of Pediatrics, National Taiwan University Hospital and College of Medicine, National Taiwan University, Taipei, Taiwan; 3 National Institute of Diagnostics and Vaccine Development in Infectious Diseases, School of Public Health, Xiamen University, Xiamen, China; 4 Department of Internal Medicine, National Taiwan University Hospital and College of Medicine, National Taiwan University, Taipei, Taiwan; Duke University, United States of America

## Abstract

**Objectives:**

Hepatitis E virus (HEV) is an emerging pathogen. We evaluated the seroprevalence of HEV infection among swine farmers and the general population in Taiwan, a nonendemic country.

**Methods:**

We conducted a cross-sectional seroepidemiologic study in rural Taiwan in 2012 and 2013. The study included swine farmers, health examination attendees, pregnant women, and students. A commercial enzyme-linked immunosorbent assay was used to detect immunoglobulin G (IgG) and IgM against HEV. Pertinent information was collected using a questionnaire.

**Results:**

In total, 660 participants were enrolled in the study, including 156 swine farmers, 314 health examination attendees, 100 pregnant women, and 90 students. IgG anti-HEV was detected in 29.5% of swine farmers, 11.5% of health examination attendees, 2% of pregnant women, and 1.1% of students. Two subjects were positive for IgM anti-HEV. Logistic regression analysis revealed that swine farmers had an approximately 3.5-fold increased risk (odds ratio [OR], 3.46; 95% confidence interval [CI], 1.91–6.27; p<0.0001) for being seropositive for IgG anti-HEV as compared to the general population. Age was positively associated with seropositive rate (OR, 1.07 per year; 95% CI, 1.05–1.09; p<0.0001).

**Conclusion:**

HEV infection is prevalent in Taiwan. The seroprevalence of HEV infection is high in swine farmers and in the elderly population.

## Introduction

Hepatitis E virus (HEV), a small non-enveloped RNA virus, is the only member of the genus *Hepevirus* in the family *Hepeviridae* [1]. It is thought to be the most common cause of acute hepatitis worldwide [2]. HEV causes more than 3 million symptomatic infections each year [3]. The major route of transmission is fecal-oral, waterborne, and foodborne [2]. HEV infections are largely asymptomatic, but can cause acute hepatitis and fulminant hepatic failure with an approximately 20% mortality rate in pregnant women living in highly endemic countries [4]. There are 4 HEV genotypes but only 1 serotype [5]. HEV genotypes 1 and 2 infect only humans and typically cause either sporadic cases or outbreaks in developing countries [6]. HEV genotypes 3 and 4 infect pigs and other mammalian animals in both developing and developed countries (autochthonous infection); humans are accidental hosts [7]. Although HEV infections in developed countries are less common, seroprevalence varies widely from 1% to >20%, depending on the diagnostic kit used for detection [8], [9]. Over the past decade, there has been an emergence of HEV infections and zoonotic transmission in developed countries, particularly in Japan [10] and Europe [11]. Chronic HEV infections have received increasing attention in recent years because they pose a threat to immunocompromised hosts, including solid-organ-transplant recipients [12], patients with hematological disorders receiving chemotherapy [13], and those with human immunodeficiency virus infections [14].

In Taiwan, endemics have occurred for hepatitis A, B, and C, but not for hepatitis E. However, sporadic and locally acquired HEV infections have been documented in Taiwan [15]; the most commonly observed HEV genotype is genotype 4. According to the Centers for Disease Control in Taiwan, the crude incidence rate of acute hepatitis E was 0.03–0.06/100,000 person-years in 2008–2012 [16]. However, an increasing number of HEV infections has been detected in nearby countries, including China [17], Hong Kong [18], and Japan [10]. Whether these infections are due to changing epidemiology or increased surveillance remains unclear, but it is important to understand the local epidemiology. The most recent seroepidemiologic study in Taiwan was published in 1994 [19]. We evaluated the current seroprevalence of HEV infection using a new assay kit.

## Materials and Methods

### Study Design and Study Area

**Figure 1:**
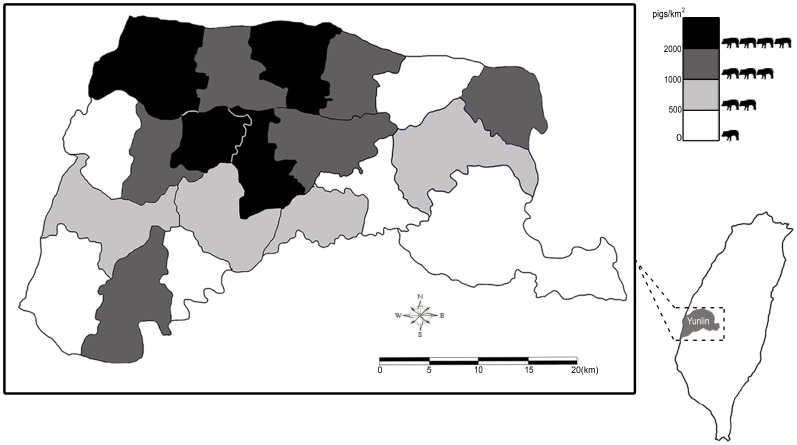
Pig density (pigs/km2) in the study area is depicted on the map (Yunlin County, Taiwan). Pig density in each township was calculated as the number of pigs divided by the size of the area.

We conducted a cross-sectional seroepidemiologic study in rural Taiwan in 2012 and 2013. Yunlin County is located in west-central Taiwan. Yunlin County has a population of 710,000 inhabitants and an area of 1291 km^2^ and is famous for its swine farming. According to the statistics compiled by the Council of Agriculture in Taiwan in 2012 [20], there were nearly 1.5 million pigs in a total of 1380 herds in Yunlin County. The pig density of Yunlin County (1153 pigs/km^2^) ranks among the highest in Taiwan (average, 173 pigs/km^2^). The pig density of the townships in Yunlin County is 154–3897 pigs/km^2^ (Fig. 1).

### Ethics Statement

This study was conducted in Yunlin County, Taiwan. Written informed consent was obtained for all subjects, and parental consent was further obtained for those aged less than 20 years. The study was approved by the Institutional Review Board of the National Taiwan University Hospital (reference number 201111038RIB).

### Recruitment of the Study Population

Swine farmers, health examination attendees, pregnant women, and students were recruited for participation in the study. We visited local swine herds to recruit swine farmers, their family members, and associated personnel working with the herds. Health examination attendees were enrolled at the National Taiwan University Hospital, Yun-Lin Branch. We enrolled pregnant women at 2 local obstetric clinics. Blood samples were taken during the first trimester and at delivery. High school students were randomly selected in the study area during routine health examination. Those who had received blood transfusion within 1 year were excluded from participation. A questionnaire was also administered to collect relevant information.

### Serological Tests for Hepatitis E Virus (HEV)

Recruited subjects underwent blood sampling, and sera were aliquoted and stored at −80°C until analysis. A commercial enzyme-linked immunosorbent assay (ELISA) from Wantai Biopharmaceutical, Inc. (Beijing, China) was used to detect immunoglobulin G (IgG) and immunoglobulin M (IgM) against HEV (anti-HEV) [21], [22]. Laboratory analysis was carried out according to the manufacturer’s instructions.

### Statistical Analysis

Data collected by the questionnaire were verified and digitalized. Univariate analysis, the chi-square test, and Fisher’s exact test were used to analyze categorical variables when appropriate. To identify independent variables associated with anti-HEV seropositivity, we performed logistic regression analysis. The magnitude of the association between variables and seropositivity is expressed as an odds ratio with 95% confidence intervals. The pig density of each township was calculated as the number of pigs divided by size of the area. We performed statistical analysis using SPSS (PASW Statistics 18.0, Chicago, IL, USA). Statistical significance was set at p<0.05.

## Results

### Study Population and Demographics

A total of 660 participants, including 156 swine farmers, 314 health examination attendees, 100 pregnant women, and 90 students were enrolled in the study. Age (years) and sex ratio (male/female) were 43.9±13.1 (2.7), 43.4±15.1 (1.4), 27.8±5.0 (0), and 12.9±1.4 (1.0) for each group, respectively. Demographic details are shown in Table 1. Swine farmers were recruited from 21 local swine herds. Most of the pregnant women entered the study at 2 months of gestation, and 9 (9%) did not complete the pregnancy for various reasons.

**Table 1:** Demographics of the study population.

Characteristics	Swine farmers	Health examination attendees	Pregnant women	Students
N	156	314	100	90
Age (years)				
Mean ± SD	43.9±13.1	43.4±15.1	27.8±5.0	12.9±1.4
Range	10–70	17–79	15–40	11–16
Sex ratio (M/F)	114/42 (2.7)	184/130 (1.4)	0/100 (0)	44/46 (1.0)
Ground water in the household (%)	15 (9.6)	14 (4.5)	2 (2)	11 (11.1)
Undercooked pork/offal consumption (%)	51 (32.7)	37 (11.8)	17 (17)	12 (13.3)
History of hepatitis (%)	32 (20.5)	65 (20.7)	3 (3)	1 (1.1)
Travel to China (%)	33 (21.2)	33 (10.5)	1 (1)	0 (0)

N, numbers of the subjects; SD, standard deviation; M, male; F, female.

### Seroprevalence Rate of HEV Infection

Seropositivity of IgG anti-HEV was detected in 46 swine farmers (29.5%), 36 health examination attendees (11.5%), 2 pregnant women (2%), and 1 student (1.1%). The seropositive rate was significantly different between groups (p<0.0001). Seroprevalence rates stratified by age are shown in Table 2. The overall seropositive rate was 12.9% (85/660). Among the 85 individuals who were seropositive, 56 were male (66%), with a mean age of 52.4±12.4 (median, 54; range, 12–79) years. There was a trend toward seropositivity with increased age. Two subjects were positive for IgM anti-HEV, including one swine farmer and one health examination attendee.

**Table 2:** Seroprevalence of immunoglobulin G against hepatitis E virus (IgG anti-HEV).

Age (years)	Swine farmers		Health examination attendees		Pregnant women		Students		Overall	
	n/N	Rate (95% CI)	n/N	Rate (95% CI)	n/N	Rate (95% CI)	n/N	Rate (95% CI)	n/N	Rate (95% CI)
<20	0/7	0.0 (0.3–37)	0/22	0.0 (0.1–15)	0/7	0.0 (0.3–37)	1/90	1.1 (0.3–6)	1/126	0.8 (0.2–4)
21–30	1/22	4.5 (1–22)	0/46	0.0 (0–7.5)	2/65	3.0 (1–10)	–	–	3/133	2.3 (0.8–6)
31–40	6/33	18.2 (9–35)	3/76	4.0 (1–11)	0/28	0.0 (0–12)	–	–	9/137	6.6 (3.5–12)
41–50	14/43	32.6 (20–48)	8/73	11.0 (6–20)	–	–	–	–	22/116	19.0 (13–27)
51–60	17/35	48.6 (33–65)	11/51	21.6 (13–35)	–	–	–	–	28/86	32.6 (23–43)
>61	8/16	50.0 (28–72)	14/46	30.4 (19–45)	–	–	–	–	22/62	35.5 (25–48)
All	46/156	29.5 (23–37)	36/314	11.5 (8–15)	2/100	2.0 (0.6–7)	1/90	1.1 (0.3–6)	85/660	12.9 (10–16)

n denotes persons testing positive for IgG anti-HEV; N denotes persons who were tested in the respective age groups; all rates are shown as percentages; CI, confidence interval. Dash indicates that no subject was recruited in the respective age group.

### Univariate Analysis

We compared HEV seroprevalence rates between swine farmers and health examination attendees, which represented the general population. The seropositivity of IgG anti-HEV was significantly higher in subjects who were swine farmers (p<0.0001), had traveled to China (p<0.0001) and who were aged >40 years (p<0.0001). The gender (p = 0.312), ground water in the household (p = 0.202), consumption of undercooked pork or offal (p = 0.608) or a history of hepatitis (p = 0.564) had no association with HEV seropositivity.

### Multiple Logistic Regression Analysis

We included variables with significant association with HEV seropositivity for further analysis. Pig density was also included for the theoretical importance. Based on multiple logistic regression analysis (Table 3), swine farmers had an approximately 3.5-fold (odds ratio [OR], 3.46; 95% confidence interval [CI], 1.91–6.27; p<0.0001) higher risk than the general population to be seropositive for IgG anti-HEV. Age was independently associated with seropositive rate (OR, 1.07 per year; 95% CI, 1.05–1.09; p<0.0001). A travel history to China was not significantly associated with seropositive rate (OR, 1.75; 95% CI, 0.93–3.28; p = 0.084), as well as the pig density of the townships in which the subjects lived (OR, 1.08 per 1000 pigs/km^2^; 95% CI, 0.85–1.37; p = 0.533).

**Table 3:** Multivariate logistic regression analysis for risk factors associated with the seropositive rate.

Factors	OR	95% CI	p value
Swine farmers	3.46	1.91–6.27	<0.0001
Age (per year)	1.07	1.05–1.09	<0.0001
Travel to China	1.75	0.93–3.28	0.084
Pig density (per 1000 pigs/km^2^)	1.08	0.85–1.37	0.533

OR, odds ratio; CI, confidence interval.

## Discussion

Our study revealed that HEV infection is prevalent in Taiwan. The seropositive rate among the general population (12%) in rural Taiwan was comparable to that in the United Kingdom (16.2%) and Korea (23.1%), but less than that in the highly endemic southwest France (52.5%) when the same diagnostic assay was used [22]–[24]. A previous study in northern Taiwan showed a similar seroprevalence (10.7%) in 1994 [19]. However, direct comparisons between the 2 studies were limited by the use of different diagnostic assays [22]. The assay kit used in the current study is more sensitive than the HEV diagnostic serology kits previously available. In developed countries, the dynamics of HEV seroprevalence is intriguing. HEV seroprevalence in Japan remained constant between 1974 and 1994 [25], suggesting low-level exposure. Denmark experienced a decrease in seroprevalence of both HEV and hepatitis A virus (HAV) between 1983 and 2003 [26], suggesting the effectiveness of improved sanitation and the role of cohort effect. Only 1–2% of sporadic HEV infections by zoonotic genotypes 3 and 4 are symptomatic [27]. Given the low incidence of HEV infections but relatively high HEV seroprevalence in Taiwan, low exposure to HEV and subclinical HEV infections may have existed previously in Taiwan. In our study, the seroprevalence of HEV infection was higher in the elderly population. A marked decrease in HAV seroprevalence has been observed in the Taiwanese population born after 1982 [28]. Whether the high seroprevalence in the elderly population in Taiwan indicates constant exposure to HEV or if this observation was a result of the cohort effect requires additional analysis.

Compared with the general population, swine farmers had a 3.5-fold greater risk of being seropositive for HEV in our study. The association between occupational exposure to pigs and significantly higher seroprevalence of HEV had been reported previously for swine farmers in Eastern Europe [29] and for pig handlers in Taiwan [30], but not in Denmark [26] or northern Thailand [31]. These findings may be due to different epidemiology from different regions. In developed countries, pigs have been recognized as important factors in HEV transmission [2]. However, environmental exposure is also considered a potential source of HEV transmission, as revealed by the presence of HEV RNA in surface water samples in the Netherlands [32]. This may be responsible in part for the high seroprevalence in the general population.

A travel history to China was positively associated with HEV seropositivity in the univariate analysis but the factor was not significant in the multiple logistic regression analysis. Long been considered an endemic country, China continued to have occasional foodborne outbreaks in recent years, mainly caused by HEV genotype 4 [2]. According to Taiwan Centers for Disease Control, 35% of the cases (18/51) with acute hepatitis E were imported in 2008–2012 [16]. Locally acquired HEV infections remained important clinically and a lack of travel history should not preclude the possibility of hepatitis E in Taiwan. Pig density was not significantly associated with the seroprevalence rate in our study. Pigs were concentrated and reared on swine farms in the study area. Other than swine farming, people have little direct contact with pigs. Although confounding factors may exist because we did not take migration of the subjects into consideration, our results suggest that occupational exposure is more important than casual contact.

Autochthonous infections of HEV genotypes 3 and 4 were much more common in elderly men and in those with pre-existing liver diseases such as chronic hepatitis B infection [2], [8]. Despite the administration of universal hepatitis B vaccination since 1984, a considerable disease burden in the elderly population of Taiwan continues to exist. The seroprevalence rates of hepatitis B virus and hepatitis C virus were approximately 15% and 5% in persons born before 1970 in the study area [33], [34]. Although most locally acquired HEV infections are self-limited, they can cause “acute-on-chronic” liver failure and even mortality [8]. Increased surveillance of HEV infections should be considered during the follow-up of these patients. In our study, only 2% of pregnant women were seropositive for anti-HEV during the first trimester. Anecdotal reports have examined HEV genotype 3 infections during pregnancy [35]. Although the role of HEV genotype 4 infections during pregnancy was not clear, most pregnant women were found to be susceptible to HEV infections in the study area. A recombinant HEV vaccine recently became available in China [27] but further evaluation is needed to develop methods for HEV prevention in specific risk groups in different regions.

There were some limitations to this study. The sample size was relatively small and may not be fully representative of the study area. Our results may not correlate to seroprevalence in urban areas.

In conclusion, HEV infection is prevalent in Taiwan. The seroprevalence of HEV infection is higher in swine farmers and in the elderly population. The disease burden of HEV infection in patients with pre-existing liver diseases and pregnant women should be examined. Increased surveillance of specific risk groups should be conducted to understand the local epidemiology and guide vaccine strategies.
